# Prostaglandin-E_2_ levels over the course of glyceryl trinitrate provoked migraine attacks

**DOI:** 10.1016/j.ynpai.2022.100112

**Published:** 2022-12-28

**Authors:** Aster V.E. Harder, Gerrit L.J. Onderwater, Robin M. van Dongen, Marieke Heijink, Erik W. van Zwet, Martin Giera, Arn M.J.M. van den Maagdenberg, Gisela M. Terwindt

**Affiliations:** a Department of Neurology, Leiden University Medical Center, Leiden, The Netherlands; b Department of Human Genetics, Leiden University Medical Center, Leiden, The Netherlands; c Center for Proteomics and Metabolomics, Leiden University Medical Center, Leiden, The Netherlands; d Department of Biomedical Data Sciences, Leiden University Medical Center, Leiden, The Netherlands

**Keywords:** Migraine, Glyceryl trinitrate, Prostaglandin E_2_, Preictal, Plasma

## Abstract

Administration of glyceryl trinitrate (GTN), a donor of nitric oxide, can induce migraine-like attacks in subjects with migraine. Provocation with GTN typically follows a biphasic pattern; it induces immediate headache in subjects with migraine, as well as in healthy controls, whereafter only subjects with migraine may develop a migraine-like headache several hours later. Interestingly, intravenous infusion with prostaglandin-E_2_ (PGE_2_) can also provoke a migraine-like headache, but seems to have a more rapid onset compared to GTN. The aim of the study was to shed light on the mechanistic aspect PGE_2_ has in migraine attack development. Therefore, PGE_2_ plasma levels were measured towards the (pre)ictal state of an attack, which we provoked with GTN. Blood samples from women with migraine (n = 37) and age-matched female controls (n = 25) were obtained before and ∼ 140 min and ∼ 320 min after GTN infusion. PGE_2_ levels were measured using liquid chromatography tandem mass spectrometry (LC-MS/MS) analysis. Data was analyzed using a generalized linear mixed-effect model. Immediate headache after GTN infusion occurred in 85 % of migraine participants and in 75 % of controls. A delayed onset migraine-like attack was observed in 82 % of migraine subjects and in none of the controls. PGE_2_ levels were not different between the interictal and preictal state (*P* = 0.527) nor between interictal and ictal state (defined as having migraine-like headache) (*P* = 0.141). Hence, no evidence was found that a rise in PGE_2_ is an essential step in the initiation of GTN-induced migraine-like attacks.

## Highlights

• In this study attacks of migraine were provoked with GTN and levels of PGE_2_ were measured in blood over the course of an attack.
• No evidence was found that a rise in PGE_2_ is an essential step in the initiation of GTN-induced migraine attack.
• This suggests that the PGE_2_ pathway may not be a good future drug target.

## Introduction

**Fig. 1:**
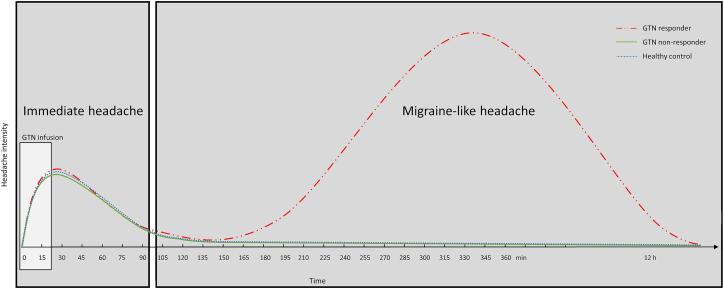
Schematic headache pattern after the start of the GTN infusion consisting of the immediate headache and the migraine-like attack. Three different response groups can be distinguished. The red two-dot chain line represents a typical headache pattern for a subject with migraine who responded to GTN (GTN responder),this is combined with typical patterns for a subject with migraine who did not respond to GTN (GTN non-responder) represented by the contineous green line, and a healthy control represented by the dotted blue line. GTN, glyceryl trinitrate. Adapted from Onderwater et al. (Onderwater et al., 2020). (For interpretation of the references to colour in this figure legend, the reader is referred to the web version of this article.)

Migraine is a common multifactorial paroxysmal brain disorder with a life-time prevalence of 15–20 %, causing disability worldwide (Gbd, 2019, Launer et al., 1999). A typical migraine attack consists of a preictal, an ictal (aura and/or headache), and a postictal (postdromal) phase (Headache Classification Committee of the International Headache Society (IHS), 2018). The pathophysiological mechanisms underlying migraine attacks, however, remain to be fully elucidated. Notably, migraine-like attacks can be induced in subjects with migraine, but not in healthy controls, by the administration of glyceryl trinitrate (GTN), a donor of nitric oxide (NO). Two types of NO-induced headaches have been reported (Fig. 1) (Olesen, 2008). First, in both migraine subjects and healthy controls an immediate headache develops within the first hour of GTN infusion. This headache is of mild to medium severity and typically resolves within an hour after GTN administration. Second, only in subjects with migraine, a delayed onset migraine-like headache (moderate to severe, accompanied by associated symptoms such as nausea, vomiting, photo- and/or phonophobia) may develop within 12 h after GTN infusion (Bagdy et al., 2010, Iversen et al., 1989). This different response to GTN in cases compared to controls may provide clues for mechanisms underlying migraine attacks. Whereas the immediate headache seems related to a direct action of the NO-cGMP pathway via vasodilation by smooth muscle relaxation (Akerman et al., 2002), independent of neuropeptide calcitonin gene-related peptide (CGRP) release (Bellamy et al., 2006), the delayed migraine-like attack is thought to be the result of trigeminovascular activation mediated via CGRP release (Akerman et al., 2002, Bagdy et al., 2010, Juhasz et al., 2003).

Besides CGRP there is ample evidence that prostaglandins may be pivotal in the development of GTN-induced migraine-like attacks, and possibly spontaneous migraine attacks (Davis et al., 2004). NO stimulates cyclooxygenase (COX-1 and COX-2) synthesis, which are enzymes that produce prostaglandins (Mollace et al., 2005). Non-steroidal anti-inflammatory drugs (NSAIDs), which inhibit prostaglandin synthesis, are a first line treatment for migraine headaches. Cortical spreading depolarization, the underlying mechanism for the migraine aura, causes COX-2 upregulation potentially leading to increased prostaglandin levels (Eikermann-Haerter and Ayata, 2010, Yokota et al., 2003). The role of prostaglandins has also been investigated in provocation experiments in migraine subjects, in most cases in those without aura, demonstrating that intravenous infusion of prostaglandin I_2_ (PGI_2_) and E_2_ (PGE_2_) induces migraine like-attacks in 75 % of participants with migraine (Antonova et al., 2012, Wienecke et al., 2010). Remarkably, subjects with migraine typically developed rapid onset migraine-like attacks, with a median onset of 20 min, in 25 % (PGI_2_) and 58 % (PGE_2_) of cases, which is in contrast to provocation with GTN, pituitary adenylate cyclase–activating peptide (PACAP) and CGRP for which the majority of cases develops a delayed onset migraine-like attack after at least a few hours (Antonova et al., 2012, Ashina et al., 2013).

**Fig. 2:**
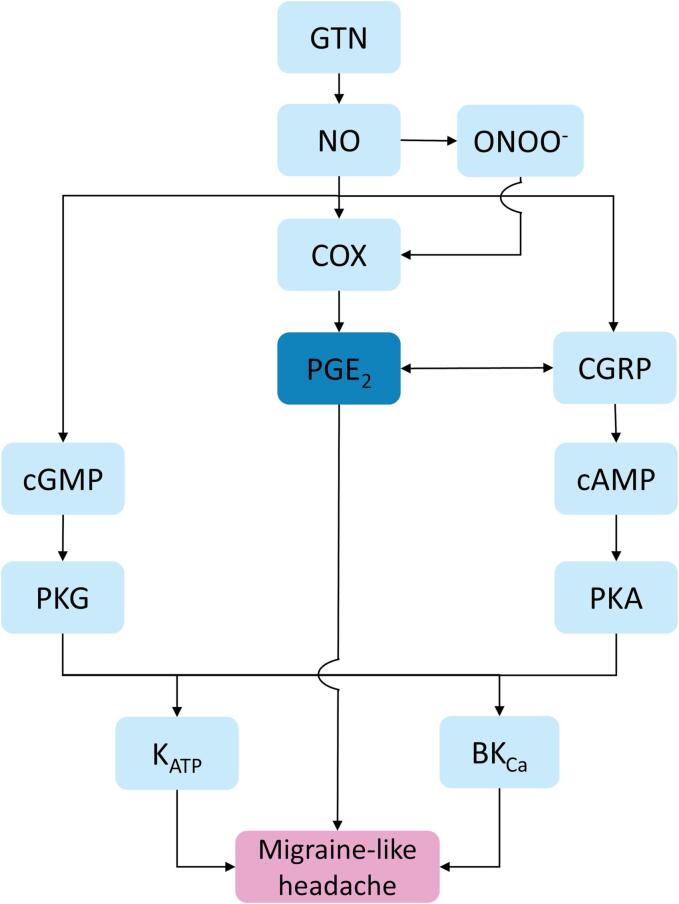
Pathway relevant to nitroglycerin (GTN)-induced migraine-like headache. Nitroglycerine (GTN) liberates nitric oxide (NO) in peripheral and cerebral structures. NO subsequently, by binding to soluble guanylyl cyclase (sGC), increases cyclic guanosine monophosphate (cGMP) (Arnold et al., 1977). Furthermore, NO can interact with superoxide to form peroxynitrite. Peroxynirite (ONOO^−^) is a proinflammatory compound and has been implicated in the pathophysiology of not only stroke, but also pain and is gaining interest in the migraine field (Bredt, 1999, Taffi et al., 2005). Additionally, NO on the one hand stimulates COX synthesis and prostaglandin E_2_ (PGE_2_) production (Mollace et al., 2005), and on the other hand stimulates CGRP, independent of the cGMP signaling pathway (Bellamy et al., 2006). Subsequently, CGRP has been shown to induce PGE_2_ (Kress et al., 1999), and vice versa (Jenkins et al., 2001, Jenkins et al., 2004, Neeb et al., 2011). In turn, it has been shown that ONOO^−^ when inducing inflammation-derived hyperalgesia acts via the COX-to-PGE_2_ pathway (Ndengele et al., 2008), and ONOO^−^ is also implicated along the trigeminovascular migraine pathway associated with CGRP (Akerman et al., 2021). PKG-mediated phosphorylation opens ATP-sensitive potassium channels (K_ATP_) channels and large (big)-conductance calcium-activated K^+^ (BK_Ca_) via the NO/cGMP/PKG pathway (Murphy and Brayden, 1995, Schubert and Nelson, 2001). CGRP activates vascular smooth muscle K_ATP_ channels and BK_Ca_ channels via cyclic adenosine monophosphate (cAMP) and protein kinase A (PKA) phosphorylation (Hosokawa et al., 2010, Miyoshi and Nakaya, 1995). PGE_2_ can also either increase or decrease the amount of cAMP depending on to which receptor it binds (Markovič et al., 2017). Opening of K_ATP_ and BK_Ca_ channels generates outward K^+^ currents and causes vasodilation (Chrissobolis and Sobey, 2003), and can eventually lead to a migraine-like attack (Al-Karagholi et al., 2021, Al-Karagholi et al., 2019). Provocation with PGE_2_ in subjects with migraine leads to a rapid-onset migraine attack (Antonova et al., 2012), which suggests that PGE_2_ is closely upstream of a migraine-like attack.

It has been shown that PGE_2_ is mediated via CGRP release, and vice versa (Davis et al., 2004), as evidenced by observations that PGE_2_ stimulates the release of CGRP in rat trigeminal neurons (Jenkins et al., 2001), trigeminal nucleus caudalis (Jenkins et al., 2004), and trigeminal ganglia (Neeb et al., 2011), while CGRP induces secondary release of PGE_2_ (Kress et al., 1999). All the above suggests that PGE_2_ may be closely upstream of GTN-induced migraine attacks (Fig. 2). We here aimed to shed light on the mechanistic aspect PGE_2_ has in migraine attack development, as it might serve as a possible drug target. We measured PGE_2_ plasma levels in female subjects with migraine and age-matched female healthy controls in the (pre)ictal phases of GTN provoked migraine-like attacks to assess whether PGE_2_ levels change as part of GTN-induced migraine attacks.

## Methods

### Participants

This study was conducted as part of an extensive migraine provocation study, described in Onderwater et al. (Onderwater et al., 2020). In total, 37 female subjects with migraine (without aura) and 25 age-matched female healthy controls were included. Due to the predominance of migraine in females only female subjects were included in the study. Migraine was diagnosed in accordance with the International Classification of Headache Disorders (ICHD-3) (Headache Classification Committee of the International Headache Society (IHS), 2018). Participants with migraine experienced one or more migraine attacks per month during the past six months. Subjects with chronic migraine or medication-overuse headache were excluded. Healthy controls were free of (severe) headaches, neurological or psychiatric disorders and had no family history of severe primary headaches, but were allowed to occasionally have tension-type headaches. None of the participants used chronic medication other than oral contraceptives. The study was approved by the ethics committee of the Leiden University Medical Center and in accordance with the World Medical Association Declaration of Helsinki. All participants provided written informed consent prior to the study.

### Study design

During the study day, each participant was subjected to detailed interviews over the course of the day and underwent three blood withdrawals. Samples were drawn by venipuncture from the medial cubital vein. Participants were attack-free at least three days prior to the investigation and had been instructed to refrain from using prophylactic medication for at least four weeks. Apart from abstaining from alcoholic beverages, caffeinated beverages, and smoking for at least 8 h prior to and during the study day there were no dietary restrictions. Before GTN infusion, all participants underwent a baseline assessment consisting of a neurological examination, headache assessment, and a blood withdrawal in ethylenediaminetetraacetic acid (EDTA)-containing tubes was performed for baseline measurement [T0]. Following the baseline measurement, participants received an intravenous infusion of GTN (0.5 µg/kg/min over 20 min) between 9:45 and 10:45 AM, in supine position. After GTN infusion, blood was again drawn from participants at two time points, namely ∼ 140 min [T1] and ∼ 320 min after the start of GTN infusion [T2]. To avoid biochemical interference in the processes related to the initiation and onset of a migraine-like headache, participants were requested to abstain from using acute migraine attack medication until after the 3rd and final blood measurement [T2]. Blood was centrifuged at room temperature for 20 min (2,000 rpm, 622 *g*). The supernatant was transferred to a 15-mL polypropylene tube (Greiner Bio-One CELLSTAR®), inverted several times, and divided in 0.5-mL aliquots (1.0 mL Nunc^TM^ cryotubes). Plasma samples were stored at − 80 °C until further use; no extra freeze–thaw cycles were allowed.

### Migraine-like headache and criteria

Participants were notified that GTN could potentially induce a headache, without any information regarding the expected onset or course. Questionnaires were performed, as described in Onderwater et al. (Onderwater et al., 2020). In short, during the 20-minute GTN infusion, headache characteristics and associated symptoms were documented every 5 min. After the infusion period, the occurrence of premonitory symptoms, headache, and associated symptoms was documented every 15 min until 5 h after GTN infusion. After the study day (6 h after GTN infusion), to determine GTN responder status, participants filled in a headache diary and were asked for headache fitting migraine-like attack onset in a telephone follow-up ∼ 3 days after participation. Headache intensity was scored with a verbal rating scale (VRS) from 0 to 10 (0 indicating no headache, 1 indicating a very mild headache and 10 indicating the worst possible headache pain imaginable). In addition, the response form included the type of pain, localization, associated symptoms, premonitory symptoms, and adverse events. Furthermore, subjects with migraine were asked whether the reported headache resembled their usual migraine attacks. Despite the resemblance with spontaneous attacks, induced attacks are referred to as ‘migraine-like headaches’, as they cannot fulfil all criteria of a migraine without aura attack; for this the attack needs to be spontaneous and last (untreated) at least 4 h (Headache Classification Committee of the International Headache Society (IHS), 2018). Therefore, in accordance with earlier provocation studies (Ashina et al., 2013), migraine-like attack onset (ictal) was determined as either (1) a moderate to severe headache (VRS ≥ 4) fulfilling ICHD-3 criteria C and D for migraine without aura or (2) a headache described as mimicking the subject’s usual migraine attack and treated with acute migraine medication.

### PGE_2_ quantification

PGE_2_ was quantified in EDTA plasma using a method analogue for the quantification of 8-*iso*-PGF2α, previously described (Dekker et al., 2019). In short, 250 μL EDTA plasma was diluted with 2.0 mL sodium acetate buffer (0.1 M, pH 3.5) and 3 μL PGE2-d4 (50 ng/mL) in methanol (MeOH) was added. The samples were loaded onto C18 SPE cartridges (200 mg, 3 cc; Waters, Sep-Pak, Milford, MA) that had been conditioned and equilibrated with MeOH and water. After a wash with water and *n*-hexane samples were eluted using methyl formate. Eluates were then dried under a gentle stream of nitrogen at 40 °C and reconstituted in 150 μL 40 % MeOH.

Samples were measured by Liquid Chromatography (Shimadzu SIL-30AC autosampler, two Shimadzu LC-30AD pumps and a Shimadzu CTO-20AC column oven) coupled to a Sciex Qtrap 6500 mass spectrometer. Forty-μL samples were injected and separated on a C18 column (Phenomenex, 50 × 2.1 mm, 1.7 µm). A gradient of 0.01 % acetic acid in water (A) and 0.01 % acetic acid in MeOH (B) was used to elute the components of interest from the column. The total flow rate was 400 μL/min. The column oven was set to 50 °C. The mass spectrometer (MS) was equipped with an ESI source and operated in negative scheduled MRM mode. The needle voltage was set to − 4,500 V, the drying temperature to 450 °C, ion source gas 1/nebulizer gas (air) at 40 psi, ion source gas 2/drying gas (air) at 30 psi and the nebulizer gas (nitrogen) at 30 psi. For PGE_2_ the transition used was 351/271, for PGE_2_-d4 355/193. PGE_2_ was identified based on its tandem MS transition and relative retention time and, quantified using external calibration.

### Statistical analysis

We aimed to investigate the role of PGE_2_ over the course of a provoked migraine attack, healthy controls were included to ensure that direct pharmacological effects of the provocation substance itself is not incorrectly labelled as a marker for provoked attacks. As we were primarily interested in the effect of different phases on PGE_2_ levels in blood, we distinguished three phases: interictal (outside a migraine-like attack), preictal (before a migraine-like headache of which the onset is ≤ 12 h after GTN infusion), and ictal (migraine-like headache). To account for repeated measurements within each subject, we used a linear mixed model with a random effect per person and unstructured correlation, the same model was used previously (Onderwater et al., 2021). The outcome (dependent variable) was the measured PGE_2_ concentration. Predictors (independent variables) were age, diagnosis (migraine or control), time point (T0, T1, T2) and migraine phase (interictal, preictal, ictal). Controls were coded as “interictal” at all time points. Furthermore, we added the interaction between time point and diagnosis to account for subjects with migraine possibly reacting differently to GTN than controls, irrespective of migraine phase. Statistical analyses were performed using SPSS (version 25.0, IBM SPSS Statistics for Windows, IBM Corp, Armonk, NY). In this study, data was collected as part of an extensive larger study and, therefore, no *a priori* power calculations were performed for this sub-study.

## Results

### Clinical characteristics

We initially included n = 37 participants with migraine and n = 25 healthy controls, of which five participants were excluded for further analyses. Two cases were removed as GTN infusion was not performed, both participants withdrew from participation after the baseline measurement. Two cases were excluded, because we were unable to classify the provoked headache attack (one not fully fulfilling a migraine-like headache nor classifying as a non-responder and the other developed a migraine-like attack, but already proceeded to a postdrome state during the study day). One healthy control was excluded due to a (first) provoked migraine-like headache. In total, data from n = 33 participants with migraine and n = 24 healthy controls were included in the analyses. The demographic and clinical characteristics of cases and controls are shown in Table 1. There were no adverse events reported.

**Table 1:** Demographic and clinical characteristics of the study population.

Participants Characteristics	Migraine cases (n = 33)	Healthy controls (n = 24)	*P* value	GTN responders (n = 27)	GTN non-responders (n = 6)
General characteristics					
Age	34.3 ± 8.2	35.2 ± 9.1	0.709^†^	35.2 ± 8.4	30.3 ± 6.4
BMI	22.9 ± 2.6	23.2 ± 2.7	0.714^†^	23.3 ± 2.7	21.6 ± 1.4
Smoking n (%)	5 (15.1 %)	3 (12.5 %)	1.000^‡^	5 (18.5 %)	0 (0 %)
Migraine characteristics					
Age of onset	16.3 ± 5.6		–	17.4 ± 4.7	11.2 ± 6.7
Migraine days (attack/month)	4.7 ± 2.7		–	5.1 ± 2.8	2.7 ± 0.8

Values are expressed as absolute values and percentage or mean ± SD., *P* values are calculated with ^†^ Student’s *t*-test, ^‡^ Fisher’s Exact Test. GTN, glyceryl trinitrate, BMI, body mass index.

### GTN response

**Fig. 3:**
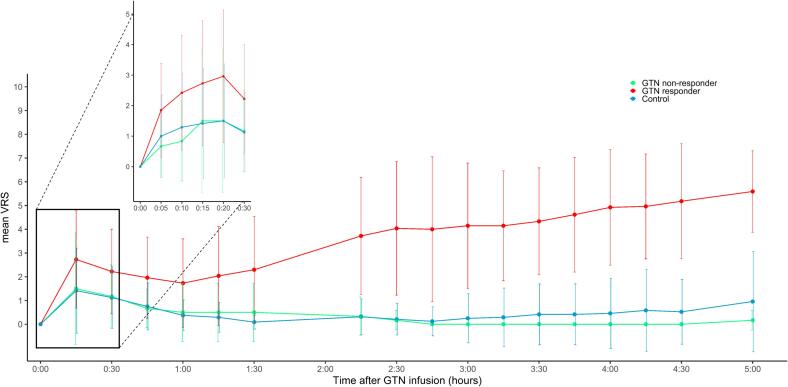
Mean verbal rating scale (VRS) for headache severity per responder group. The X-axis represents time after GTN infusion and the Y-axis the mean VRS. In red, participants with migraine who responded to GTN (GTN responders), participants with migraine who did not respond to GTN (GTN non-responders) in green and healthy controls in blue. GTN, glyceryl trinitrate; VRS, verbal rating scale. All subjects (including those without headache) were used in the calculation of the average. For some time points there were many missing values, this resulted in exclusion of these time points from the figure. Whiskers represent the standard deviation from the mean. (For interpretation of the references to colour in this figure legend, the reader is referred to the web version of this article.)

**Fig. 4:**
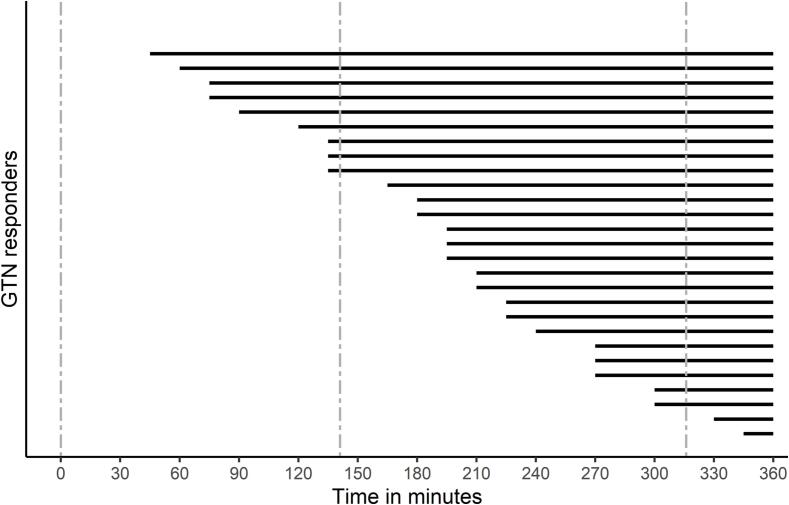
Timing migraine onset in GTN responders. The onset of migraine is plotted for each glyceryl trinitrate (GTN) responder with respect to time after GTN infusion. The start of the black continuous line represents the timing of onset of migraine attack per individual. The dotted line represents the blood draw timepoints T0, T1 and T2 at 0, ∼140 and ∼ 320 min, respectively, after the start of the GTN infusion.

In total, n = 28 subjects with migraine (85 %) and n = 18 healthy controls (75 %) developed an immediate headache (VRS ≥ 1) during the GTN infusion. At 5 min after the start of GTN infusion, the mean VRS value was 1.6 for those with migraine (1.8 for responders and 0.7 for non-responders) and 1 for controls. In total, n = 20 subjects with migraine (61 %) and n = 10 healthy controls (42 %) had an immediate headache. The mean VRS value increased until the end of the GTN infusion to 2.6 (3 for responders and 1.5 for non-responders) and 1.5, for subjects with migraine and controls, respectively. At 20 min, 26 subjects with migraine (79 %) and 13 controls (54 %) experienced a headache. Overall, the immediate headache was mild to moderate in severity and generally resolved rapidly after termination of the infusion (Fig. 3**,** Fig. S1). In some subjects with migraine a ‘’headache-free’’ interval was absent (Fig. S1), in those subjects the headache continued after infusion and eventually became more severe with characteristics of a migraine-like attack. The mean VRS for those who responded to GTN (responders) continued to increase, as the headache became more severe although only at a later stage met the criteria of migraine and in those who classified as non-responders the headache severity decreased. Generally, the immediate phase is considered to be 0–90 min post infusion. Four subjects developed migraine within this timeframe. One subject with migraine developed a headache fulfilling the migraine-like criteria within one hour after the start of GTN infusion, one at 60 min, and two at 75 min. Eventually, 27 (82 %) subjects with migraine receiving GTN experienced a migraine-like attack (Fig. 4) during the study day and 6 (18 %) did not experience such an attack, hence they were labelled as GTN responders and GTN non-responders, respectively (Table 1). Migraine-like attack onset ranged between 45 and 345 min (mean 192 ± 84 min) (Fig. 4).

### PGE_2_ in relation to migraine-like attack onset

The level of PGE_2_ per individual varied per time point (Table 2). To determine whether PGE_2_ levels were linked to the various phases (baseline, preictal and ictal) of a migraine attack, a generalized linear mixed model was used. The transition from an interictal state towards a migraine-like attack had no influence on PGE_2_ concentration (F (2, 69.70) = 1.235, *P* = 0.297). Both the transition from “interictal to preictal” (*P* = 0.527) and “interictal to ictal” (*P* = 0.141) phase of GTN-induced migraine-like attacks had no influence on PGE_2_ concentration (Table S1).

**Table 2:** Median PGE_2_ concentrations over time independent of migraine phase.

Group	[T0]	[T1]	[T2]
GTN responders	0.044 (0.02–0.10)	0.053 (0.03–0.10)	0.049 (0.03–0.08)
GTN non-responders	0.052 (0.01–0.09)	0.031 (0.01–0.07)	0.040 (0.02–0.07)
Controls	0.044 (0.02–0.08)	0.043 (0.03–0.09)	0.060 (0.03–0.09)

Values are the uncorrected medians of absolute concentrations in ng/mL with their interquartile range.

[T0] = baseline, [T1] = ∼140 min after the start of GTN infusion, [T2] = ∼320 min after GTN infusion. GTN responder, migraine patients who responded to GTN; GTN non-responder, migraine patients who did not respond to GTN. GTN, glyceryl trinitrate.

## Discussion

We performed a GTN provocation study in subjects with migraine and healthy controls and found that 82 % of migraine participants developed a delayed onset migraine-like attack. We prospectively assessed PGE_2_ levels at three time points selected over the course of provoked migraine-like attacks and compared these to those without provoked attacks and controls. We found no evidence that GTN-induced migraine-like headaches are characterized by changes in plasma PGE_2_ levels towards the (pre)ictal state. This suggests that a rise in PGE_2_ is not an essential step in the initiation of GTN-induced migraine-like attacks.

PGE_2_ is able to induce rapid-onset migraine-like attacks in subjects with migraine within 90 min (Antonova et al., 2012), in contrast to provocation with substances such as PACAP, CGRP and GTN that result in a delayed (after a few hours) onset of a migraine-like attack (Antonova et al., 2012, Ashina et al., 2013). Thus, we hypothesized that PGE_2_ could be one of the molecules involved in a(n experimentally induced) migraine attack. Given that administration of PGE_2_ can cause a rapid-onset migraine-like attack, in contrast to the other provocative substances, PGE_2_ may perhaps serve as a marker for upcoming migraine attacks, albeit that the timing of blood sampling is important. In our study, we used the GTN provocation model to assess the role of PGE_2_. It has been hypothesized that the time it takes to develop delayed migraine-like attack is due to various processes that include the regulation of gene expression and proteins ultimately resulting in migraine-like attacks in subjects with migraine with a median attack onset of 3 to 6 h, after infusion of the provocation substance. Afterall, in animal models of migraine, GTN activates the COX-2-PGE_2_ pathway in the brainstem not before 4 h after GTN administration (Tassorelli et al., 2007). However, based on our proposed mechanism and the PGE_2_ human provocation studies with rapid onset of provoked migraine-like headaches, we expected a rise in PGE_2_ to be close to the start of a migraine attack as an early marker of migraine, which would fit our time points of blood withdrawal. The alternative explanation that we did not find a rise in PGE_2_ levels might indicate that the pathway activated by GTN towards a migraine-like attack does not primarily act via PGE_2_. One can envisage that pathways, independent of PGE_2_ via for instance cGMP or cAMP, are more strongly activated than the PGE_2_-pathway when GTN is administered. Another explanation might be that a rise in PGE_2_ is very locally and hence not measurable in blood.

To our knowledge no other study measured PGE_2_ levels over the course of GTN-induced migraine-like attack in subjects with migraine. Still, few studies reporting measurements of PGE_2_ levels during spontaneous migraine attacks suggested those to be elevated in blood (Nattero et al., 1989, Sarchielli et al., 2000), and saliva (Vardi et al., 1983). More specifically, in contrast to our study, a much smaller study of only five subjects with migraine reported an increase in PGE_2_ levels in jugular venous blood peaking between 2 and 6 h after the start of a spontaneous migraine attack and normalizing towards the end of the attack (Sarchielli et al., 2000). In our study the mean attack onset was ∼ 192 min, hence many cases were over 2 h into their delayed migraine-like attack at the ∼ 320-minute time point, which suggests that our timing was not different from the spontaneous migraine attack study and thus could have picked up a similar rise in PGE_2_ levels. In addition, two studies found that PGE_2_ levels in plasma (Nattero et al., 1989), (18 cases and 12 controls) and saliva (Vardi et al., 1983) (6 cases and 9 controls) in subjects with migraine were lower compared to controls outside attacks and increased during a spontaneous attack surpassing the levels found in controls. Although this was not our primary question, we tested this and did not find a difference in baseline PGE_2_ levels between cases and controls. Giving the small number of participants in previous studies, our larger study should have been able to reveal differences in PGE_2_ levels during GTN-induced migraine-like attacks. Another reason for the discrepancy with earlier studies might be in the measuring techniques used and/or the matching and correction of data. For our study we used a highly reliable, standardized technique for measuring PGE_2_ levels and additionally have minimized external effects on PGE_2_ levels, by careful matching and correcting for multiple factors to single out the effect of PGE_2_ on a migraine attack. Whereas such external effects do not seem to have affected our results, they might have played a role in earlier studies. Another possibility is that spontaneous attacks are not always the same as provoked attacks (e.g. GTN provocation in migraine patients with aura leads to a migraine-like attack, but not an aura). This may indicate that in spontaneous attacks different pathways may be initiated depending on headache (sub)type, none the less these pathways ultimately lead to the same migraine headache.

We envisage several possible explanations why we found no evidence for a change in PGE_2_ levels over the course of a GTN-induced attack. PGE_2_ acts via four distinct G protein–coupled receptors EP1, EP2, EP3 and EP4. Ligand binding to the different EP receptors leads to the activation of distinct downstream signaling pathways, resulting in distinct biological outcomes (Markovič et al., 2017, Negishi et al., 1995), one of these second messengers being cyclic adenosine monophosphate (cAMP) (Markovič et al., 2017). Via its receptors, PGE_2_ is known to play a role in nociceptive pain processing and inflammation (Kawabata, 2011, Uda et al., 1990), exerting both damaging pro-inflammatory and protective anti-inflammatory effects in the brain (Andreasson, 2010, Liang et al., 2008, Pradhan et al., 2017). Thus, the PGE_2_ response is dependent on the array of receptors cells express as well as on intracellular pathways to which they are coupled (Andreasson, 2010, Tilley et al., 2001). Hence, any involvement of PGE_2_ in the pathogenesis of migraine may be very complex.

As mentioned previously, the immediate headache is thought to be the result of vasodilation via the NO-cGMP pathway (Akerman et al., 2002), independent of CGRP release (Bellamy et al., 2006), whereas the delayed migraine-like attack is thought to be the result of trigeminovascular activation mediated via CGRP (Akerman et al., 2002, Bagdy et al., 2010, Juhasz et al., 2003). However, there likely is extensive cross talk between both pathways (for details see Fig. 2). For instance, on a cellular level multiple components in the migraine pathway are known to be vasodilators, but can also lead to migraine attacks. As exemplified by ATP-sensitive potassium (K_ATP_) channel openers (levcromakalim) and (big)-conductance calcium-activated K^+^ (BK_Ca_) channel opener (MaxiPost), both activated via the NO-cGMP pathway, which is known to play a role in the immediate headache, but activation of these channels can also induce migraine-like attacks (Al-Karagholi et al., 2021, Al-Karagholi et al., 2019). However, the rather long delay of several hours between infusion of levcromakalim/MaxiProst and the occurrence of a migraine-like attack (with a median time of 3 h) indicates that various mediators must be involved in slower cascades of events leading to a migraine-like attack. Evidence for cross talk is that both the administration of CGRP (pathway via cAMP) and sildenafil (pathway via cGMP) can lead to a migraine-like attack, suggesting that convergence to a common cellular determinator seem to exist ultimately triggering similar attacks (Younis et al., 2019). Given that the median time until an attack for CGRP is ∼ 165 min, so much shorter than the ∼ 285 min for sildenafil, one can envisage that CGRP acts more downstream in the generation of a migraine attack. Such sequential actions, given the cross talk between CGRP and PGE_2_, especially in GTN-induced attacks, seems in line with a chain-of-events-pathway (Fig. 2).

Our study has several limitations. We collected a large number of blood samples of migraine participants and healthy controls. Each participant was sampled at three fixed times during the study day in an attempt to measure PGE_2_ concentrations during attack development and during the attack itself. We find that the 95 % confidence interval of the change in PGE2 levels from interictal to another phase extends from − 0.02 to 0.05 ng/mL. Of course, the onset of the attack varies between subjects and did not align perfectly with the measurement times. Moreover, we must account for a possible temporal effect of the GTN infusion on PGE_2_ concentrations. Combined with within and between subject measurement variation, we must acknowledge that not finding a statistically significant difference in PGE_2_ levels over the course of an induced migraine-like attack does not prove the absence of such an effect. There may yet exist subtle, short-duration, variations in PGE_2_ levels that we could not detect. Furthermore, we have used LC-MS/MS which is distinct from the more often used ELISA kits to measure PGE_2_, this might make it difficult to compare absolute concentrates between studies. However, by using this method we were able to detect very low levels of PGE_2_ with good accuracy, despite the short half-life of PGE_2_. Furthermore, whereas we did not observe changes in PGE_2_ levels in blood it is conceivable that levels may be different in cerebrospinal fluid, as increased PGE_2_ levels have been reported indicative for probable Alzheimer’s disease (Montine et al., 1999). However, we deem it too unethical and unlikely that subjects with migraine (and controls) are willing to participate in a provocation study with, logically, repeated lumbar punctures to get information on PGE_2_ levels over time. Finally, our study only consists of females to prevent any sex effects, which may limit the generalizability of our findings to male migraine patients. Additionally, although we have performed our study in a female only population to account for the most notable sex hormone differences, small differences in cycle and use of contraceptives might be of influence in the downstream provocation pathways.

## Funding information

This study was supported by the Netherlands Organization for Health Research and Development (ZonMw) (Clinical Fellowship 90700217 and VIDI grant 917.11.31 to G.M.T.), and European Community (EC) FP7-EUROHEADPAIN (602633 to A.M.J.M.v.d.M.).

## CRediT authorship contribution statement

**Aster V.E. Harder:** Conceptualization, Data curation, Formal analysis, Investigation, Writing – original draft, Methodology, Project administration. **Gerrit L.J. Onderwater:** Conceptualization, Data curation, Formal analysis, Investigation, Methodology, Project administration, Writing – review & editing. **Robin M. van Dongen:** Conceptualization, Investigation, Writing – review & editing. **Marieke Heijink:** Methodology, Validation, Writing – review & editing. **Erik W. van Zwet:** Formal analysis, Formal analysis, Writing – review & editing. **Martin Giera:** Conceptualization, Resources, Validation, Writing – review & editing. **Arn M.J.M. van den Maagdenberg:** Conceptualization, Funding acquisition, Resources, Supervision, Writing – review & editing. **Gisela M. Terwindt:** Conceptualization, Funding acquisition, Resources, Supervision, Writing – review & editing.

## Declaration of Competing Interest

The authors declare that they have no known competing financial interests or personal relationships that could have appeared to influence the work reported in this paper.

## Data Availability

Data not published within the article is available from the corresponding author upon reasonable request.
