# Supplementary material for: Prostaglandin-E2 levels over the course of glyceryl trinitrate provoked migraine attacks
Source: Neurobiol Pain. 2022 Dec 28;13:100112. doi: 10.1016/j.ynpai.2022.100112 (PMC9829921; doi:10.1016/j.ynpai.2022.100112)
Supplement: Supplementary data 1 [file mmc1.pdf]

Supplementary Fig 1

A

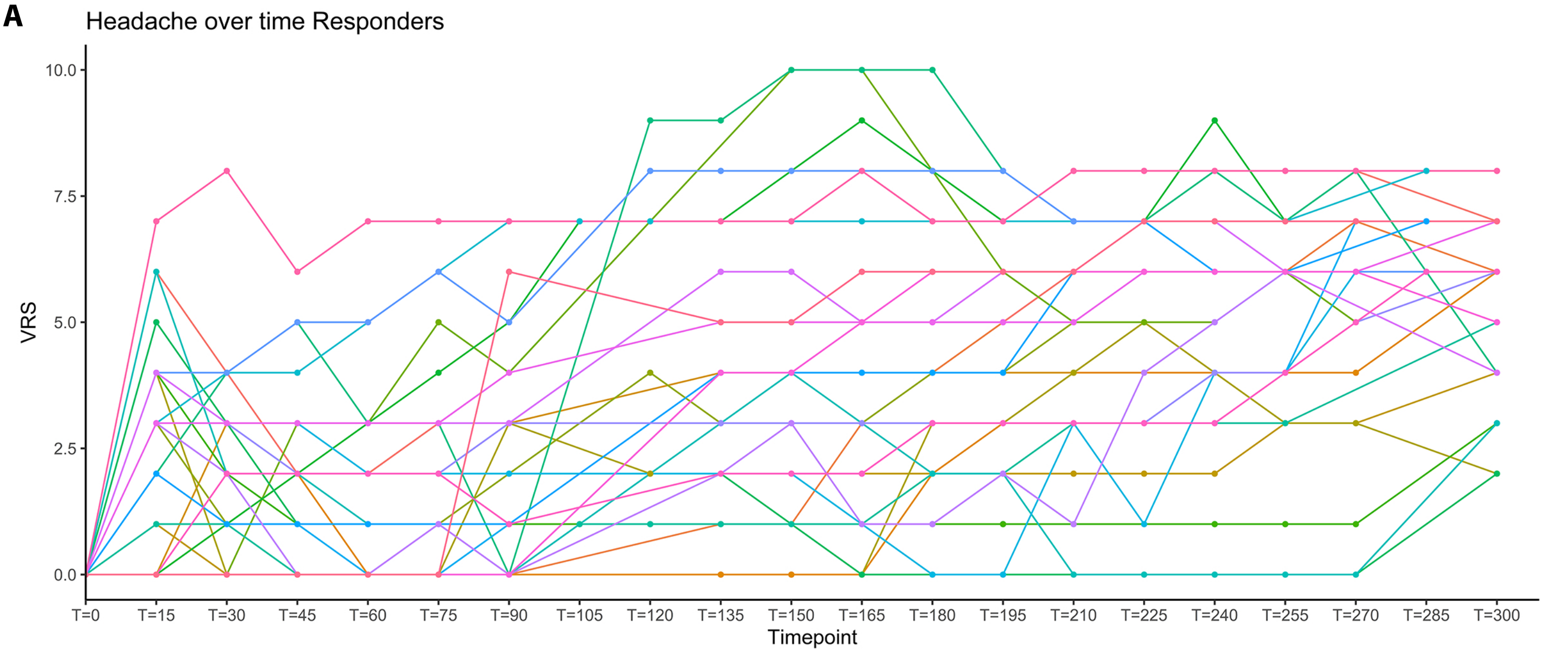

B

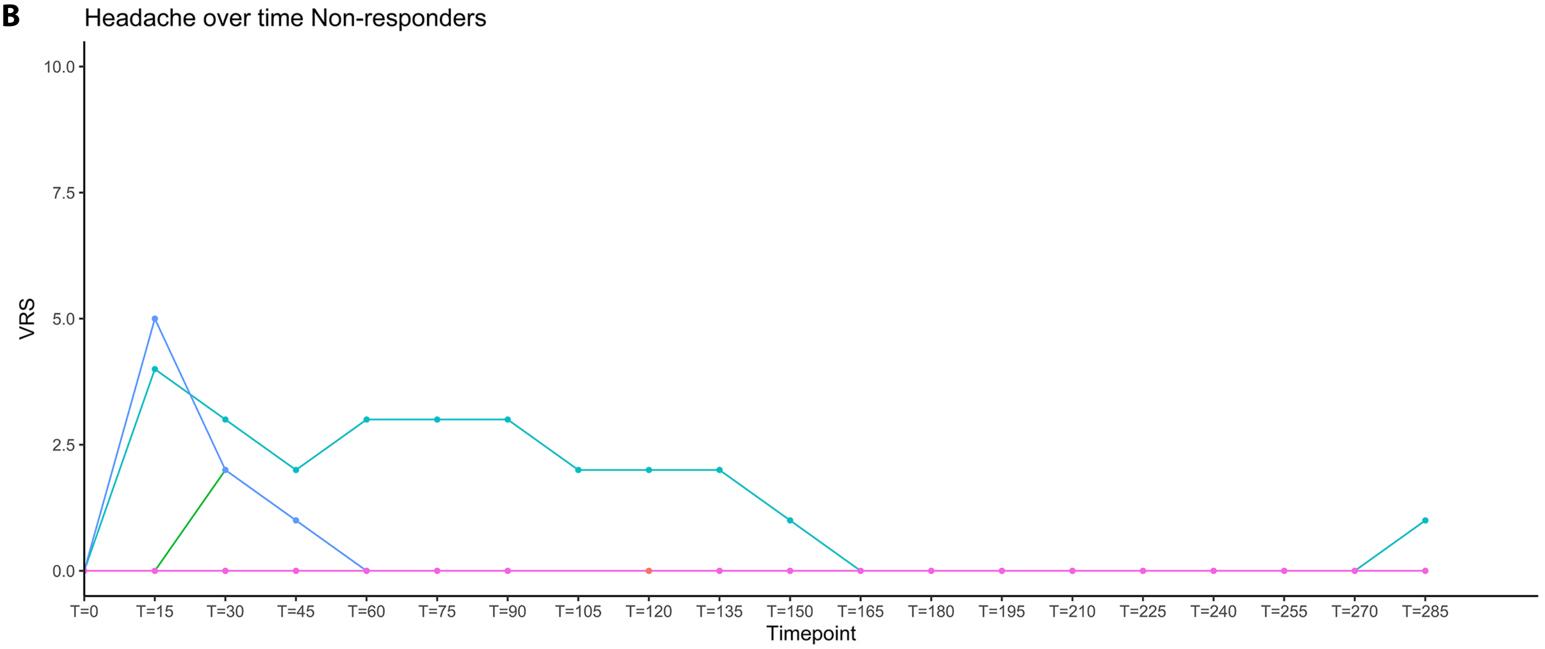

C

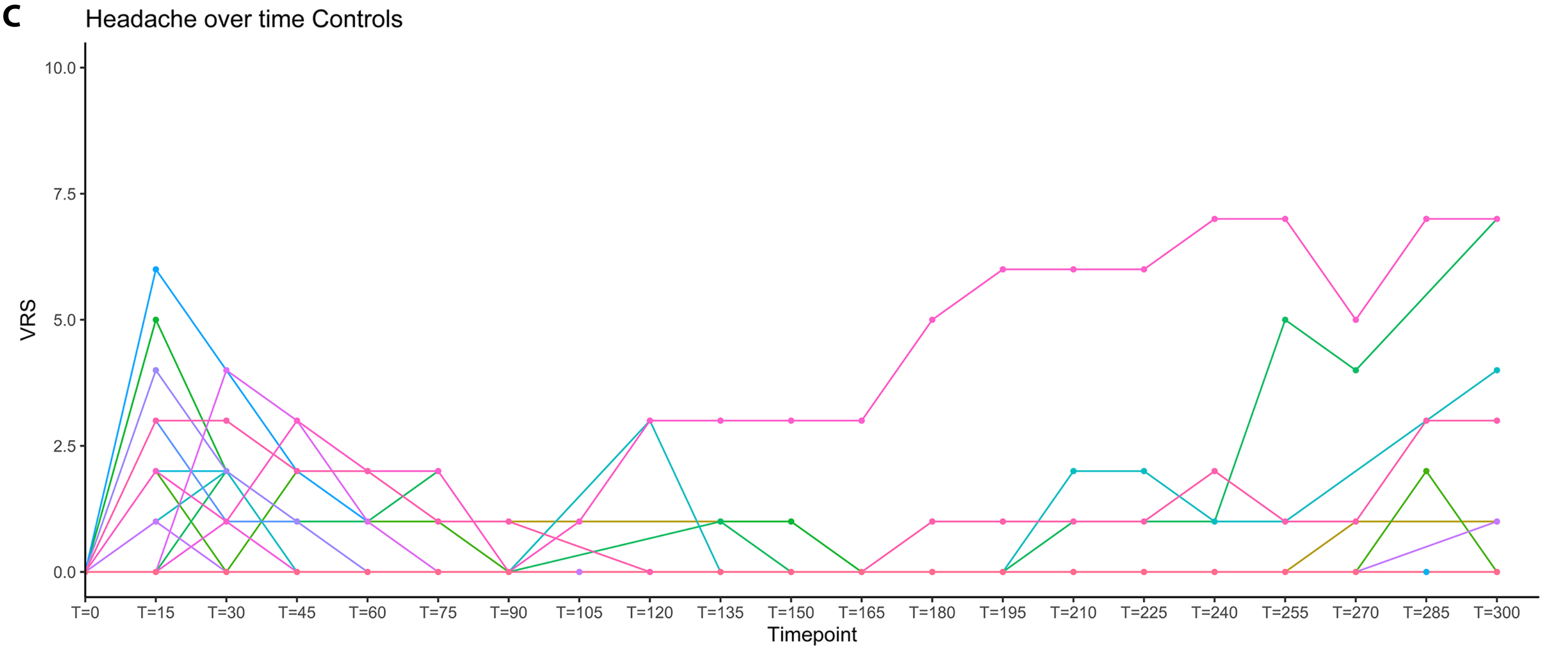

Verbal rating scale (VRS) for headache severity per individual over time. **A** Individual VRS is depicted for the 27 participants with migraine who developed a migraine-like attack after glyceryl trinitrate (GTN responders; individual cases = colored lines with dots) **B** Individual VRS is depicted for the 6 participants with migraine who did not develop a migraine-like attack after glyceryl trinitrate (GTN non-responders; individual cases = colored lines with dots). **C** Individual VRS is depicted for the 24 healthy controls after glyceryl trinitrate infusion (individual cases = colored lines with dots).
