# Supplementary material for: Prostaglandin-E2 levels over the course of glyceryl trinitrate provoked migraine attacks
Source: Neurobiol Pain. 2022 Dec 28;13:100112. doi: 10.1016/j.ynpai.2022.100112 (PMC9829921; doi:10.1016/j.ynpai.2022.100112)
Supplement: Supplementary data 2 [file mmc2.docx]

**Supplementary Table 1.** Linear mixed-effect model correction factors for PGE_2_

|  | Estimate | SE | df | t | *P* | 95%CI  lower | 95%CI  upper |
| --- | --- | --- | --- | --- | --- | --- | --- |
| Intercept | 0.122 | 0.022 | 63.18 | 5.649 | **0.000** | 0.079 | 0.165 |
| Phase (Interictal) | 0 | 0 |  |  |  |  |  |
| Phase (Preictal) | 0.010 | 0.016 | 66.92 | 0.635 | 0.527 | -0.022 | 0.043 |
| Phase (Ictal) | 0.022 | 0.015 | 57.746 | 1.491 | 0.141 | -0.007 | 0.051 |
| Diagnosis (case) | 0 | 0 |  |  |  |  |  |
| Diagnosis (control) | -0.008 | 0.014 | 54.510 | -0.583 | 0.563 | -0.036 | 0.020 |
| Timepoint (T0) | 0 | 0 |  |  |  |  |  |
| Timepoint (T1) | -0.015 | 0.015 | 72.373 | -1.043 | 0.300 | -0.045 | 0.014 |
| Timepoint (T2) | -0.021 | 0.014 | 72.377 | -1.418 | 0.160 | -0.049 | 0.008 |
| Age | -0.002 | 0.001 | 53.680 | -2.938 | **0.005** | -0.003 | -0.001 |
| Timepoint x Status (T0 x Migraine) | 0 | 0 |  |  |  |  |  |
| Timepoint x Status (T1 x Migraine) | 0 | 0 |  |  |  |  |  |
| Timepoint x Status (T2 x Migraine) | 0 | 0 |  |  |  |  |  |
| Timepoint x Status (T0 x Control) | 0 | 0 |  |  |  |  |  |
| Timepoint x Status (T1 x Control) | 0.019 | 0.017 | 72.615 | 1.052 | 0.296 | -0.017 | 0.054 |
| Timepoint x Status (T2 x Control) | 0.030 | 0.018 | 72.436 | 1.698 | 0.094 | -0.005 | 0.065 |

*P* values < 0.05 in bold. Estimate = Effect estimate, SE = standard error, df = degrees of freedom, t = t value, *P* = P value, 95%CI = 95 % confidence interval
